# Occurrence of cold sensitivity in carpal tunnel syndrome and its effects on surgical outcome following open carpal tunnel release

**DOI:** 10.1038/s41598-020-70543-8

**Published:** 2020-08-10

**Authors:** Malin Zimmerman, Erika Nyman, Lars B. Dahlin

**Affiliations:** 1 Department of Translational Medicine – Hand Surgery, Lund University, Skåne University Hospital, Jan Waldenströms gata 5, 205 02 Malmö, Sweden; 2 Department of Hand Surgery, Skåne University Hospital, Jan Waldenströms gata 5, 205 02 Malmö, Sweden; 3 Department of Biomedical and Clinical Sciences, Linköping University, Linköping, Sweden; 4 Department of Hand Surgery, Plastic Surgery and Burns, Department of Biomedical and Clinical Sciences, Linköping University Hospital, Linköping, Sweden

**Keywords:** Neuroscience, Physiology, Diseases, Neurology, Signs and symptoms

## Abstract

Cold sensitivity is common following nerve injuries in the upper extremity, but is less well studied in carpal tunnel syndrome (CTS). We investigated cold sensitivity in CTS and its effects on surgical outcome. A search of the Swedish National Registry for Hand Surgery (HAKIR) for open carpal tunnel releases (OCTR) from 2010–2016 identified 10,746 cases. Symptom severity questionnaires (HQ-8; HAKIR questionnaire 8, eight Likert-scale items scored 0–100, one item on cold sensitivity) and QuickDASH scores before and after surgery were collected. Patient mean age was 56 ± SD 16 years, and 7,150/10,746 (67%) were women. Patients with severe cold sensitivity (defined as cold intolerance symptom severity score > 70; n = 951), scored significantly higher on QuickDASH at all time points compared to those with mild cold sensitivity (cold intolerance symptom severity scores ≤ 30, n = 1,532); preoperatively 64 [50–75] vs. 40 [25–55], at three months 32 [14–52] vs. 18 [9–32] and at 12 months 25 [7–50] vs. 9 [2–23]; all p < 0.0001. Severe cold sensitivity predicted higher postoperative QuickDASH scores at three [12.9 points (95% CI 10.2–15.6; p < 0.0001)] and at 12 months [14.8 points (11.3–18.4; p < 0.0001)] compared to mild cold sensitivity, and adjustment for a concomitant condition in the hand/arm, including ulnar nerve compression, did not influence the results. Cold sensitivity improves after OCTR. A higher preoperative degree of cold sensitivity is associated with more preoperative and postoperative disability and symptoms than a lower degree of cold sensitivity, but with the same improvement in QuickDASH score.

## Introduction

Cold sensitivity is a disabling symptom that often presents after hand injuries, particularly those involving peripheral nerves in the upper extremity. It is also common, but often overlooked, in nerve compression syndromes. Cold sensitivity is defined as an abnormal response to cold, with symptoms such as numbness, pain, stiffness, weakness, and sometimes changes in colour^1^. Cold sensitivity has also been associated with poorer sensory function following nerve compression^2^, and with reduced quality of life^3^.

In cases of nerve compression syndromes in the upper extremity, earlier studies have described cold sensitivity as occurring more commonly in patients with diabetes and in women^4^. Cold sensitivity largely resolves following open carpal tunnel release (OCTR), but may persist longer in patients with diabetes than in those without^5^.

The population numbers in previous studies on cold sensitivity in nerve compression syndromes were limited. The impact of cold sensitivity on surgical outcome has not yet been completely clarified^5,6^. Hence, our aim was to investigate the occurrence of cold sensitivity in carpal tunnel syndrome (CTS) both pre- and post-operatively, and its effects on the surgical outcome of OCTR.

## Results

The study population has been described previously^7^. During the study period, 10,770 cases identified in the registry were treated with OCTR for primary CTS. Of these, 22 were excluded, being under the age of 18 years, and in two other cases the HAKIR data proved to be incorrect. Bilateral OCTR was performed in 1,717 patients during the study period. In total, this resulted in the inclusion of 10,746 cases from 9,029 patients. There were 7,150/10,746 (67%) women and mean age for both sexes at surgery was 56 ± SD 16 years. Diabetes was present in 1,509/10,746 (14%) of the cases. The most common concomitant procedures were trigger finger release (421/10,746 cases), ulnar nerve decompression at the elbow (284/10,746 cases), tenosynovectomy (211/10,746 cases) and ganglion excision (90/10,746 cases). In our cohort, 6,125/10,746 (57%) surgeries were performed during the winter period (October–March) and 4,621/10,746 (43%) during the summer period (April–September).

### Non-responders to QuickDASH and HQ-8

Response rates were 3,597/10,746 (33%) preoperatively, 2,824/10,010 (28%) at 3 months postoperatively and 2,037/8,297 (25%) at 12 months postoperatively. We found no differences between responders and non-responders regarding gender distribution, or prevalence of diabetes. However, responders were slightly older than non-responders (data already published^7^).

### HQ-8

Cold sensitivity scores improved over time, as did the other symptoms scored in the HQ-8 (Table 1). Numbness/tingling in fingers was the symptom that was scored highest by the population, particularly preoperatively (Table 1).

**Table 1:** HAKIR questionnaire 8 (HQ-8) over time in cases treated with open carpal tunnel release for carpal tunnel syndrome.

HQ-8 items	Preoperative (n = 3,327)	3 months postoperative (n = 2,563)	12 months postoperative (n = 1,987)	P-value
Cold sensitivity	50 [10–79]	3 [0–30]^a^	4 [0–30]^ns^	**< 0.0001**
Weakness	50 [27–70]	29 [10–50]^a^	20 [0–40]^c^	**< 0.0001**
Stiffness	40 [10–60]	12 [1–34]^a^	10 [0–30]^c^	**< 0.0001**
Pain on load	57 [28–78]	30 [10–50]^a^	10 [0–40]^c^	**< 0.0001**
Pain on motion without load	40 [10–60]	10 [0–26]^a^	2 [0–20]^b^	**< 0.0001**
Pain at rest	40 [17–70]	3 [0–20]^a^	1 [0–20]^ns^	**< 0.0001**
Numbness/tingling in fingers	80 [60–90]	5 [0–30]^a^	10 [0–30]^ns^	**< 0.0001**
Ability to perform daily activities	50 [30–70]	15 [1–40]^a^	10 [0–30]^c^	** < 0.0001**

Numbers presented as median [IQR].

Bold values indicate P-value < 0.05.

^a^p < 0.0001 between preoperative and 3 months postoperative values.

^b^p < 0.05 between 3 months postoperative and 12 months postoperative values.

^c^p < 0.0001 between 3 months postoperative and 12 months postoperative values.

*ns* non-significant, *IQR* interquartile range. Friedman’s two-way analysis adjusted with the Bonferroni correction for multiple testing was used for significance testing.

### Cold sensitivity and QuickDASH

Men and women reported equal frequencies of mild (HQ-8 score ≤ 30), moderate (HQ-8 score 31–70) and severe (HQ-8 score > 70) cold sensitivity (Table 2). We found no age differences between cold sensitivity groups (Table 2). Scoring of cold sensitivity did not differ between patients with diabetes and patients without diabetes (data already published^7^). HbA1c values did not differ between patients with diabetes and moderate or severe cold sensitivity and patients with diabetes and mild cold sensitivity (data not shown). Preoperative cold sensitivity correlated moderately with preoperative total QuickDASH score (Pearson 0.42; p < 0.0001).

**Table 2:** Characteristics and QuickDASH results in cases with mild (cold sensitivity symptom scoring ≤ 30), moderate (cold sensitivity symptom scoring 31–70) and severe (cold sensitivity symptom scoring > 70) cold sensitivity treated with open carpal tunnel release for carpal tunnel syndrome.

Characteristic and QuickDASH	Mild cold sensitivity (n = 1,377)	Moderate cold sensitivity (n = 1,110)	Severe cold sensitivity (n = 951)	P-value
Gender, female	913 (66)	714 (64)	630 (66)	0.4
Age, years	56 [44–67]	56 [45–68]	56 [46–68]	0.2
Diabetes	173 (13)	156 (14)	137 (14)	0.4
Preop QuickDASH	40 [25–55]	53 [39–66]*	64 [50–75]*	**< 0.0001**
QuickDASH 3 m postop	18 [9–32] (n = 518)	20 [9–39]* (n = 399)	32 [14–52]* (n = 327)	**< 0.0001**
QuickDASH 12 m postop	9 [2–23] (n = 350)	18 [5–37]* (n = 280)	25 [7–50]* (n = 224)	**< 0.0001**
Change in QuickDASH 0–12 months	24 [11–36] (n = 345)	25 [11–40] (n = 276)	26 [9–48] (n = 218)	0.11

Values presented as n (%) or median [IQR]. Groups based on how patients scored the item regarding cold sensitivity in the preoperative HAKIR questionnaire 8 (HQ-8), scored 0–100.

Bold values indicate P-value < 0.05.

*IQR* interquartile range, *preop* preoperative, *3 m postop* 3 months postoperative, *12 m postop* 12 months postoperative. Kruskal–Wallis was used for significance testing for continuous variables and Chi-Square for nominal variables.

*Significant at < 0.05 level compared to the group to the left.

**Figure 1:**
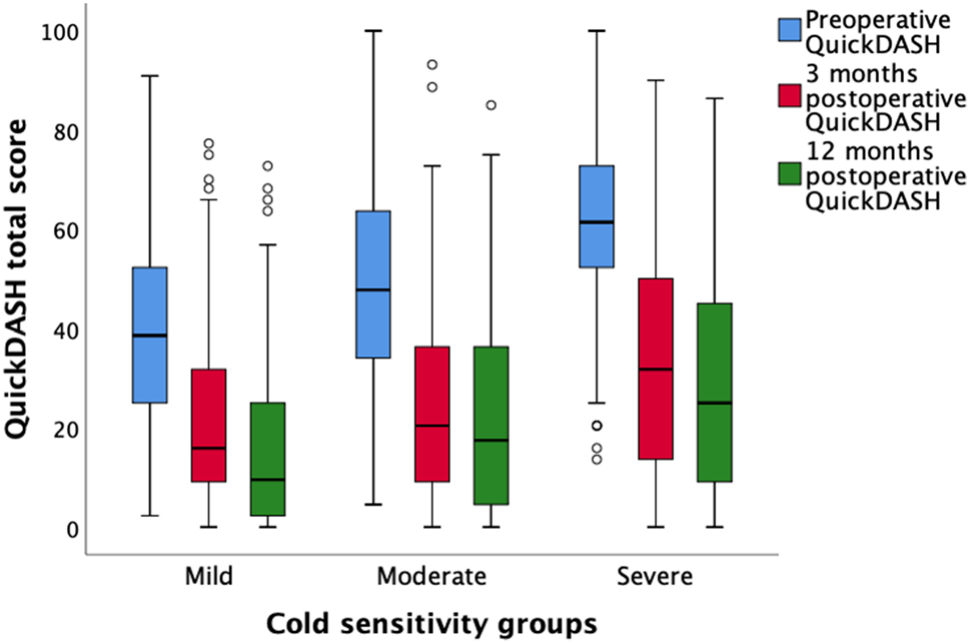
Boxplot of QuickDASH scores over time in patients with mild (scored as ≤ 30/100), moderate (scored as 31–70/100) and severe (scored as > 70/100) cold sensitivity with carpal tunnel syndrome (CTS) treated with open carpal tunnel release (OCTR).

**Figure 2:**
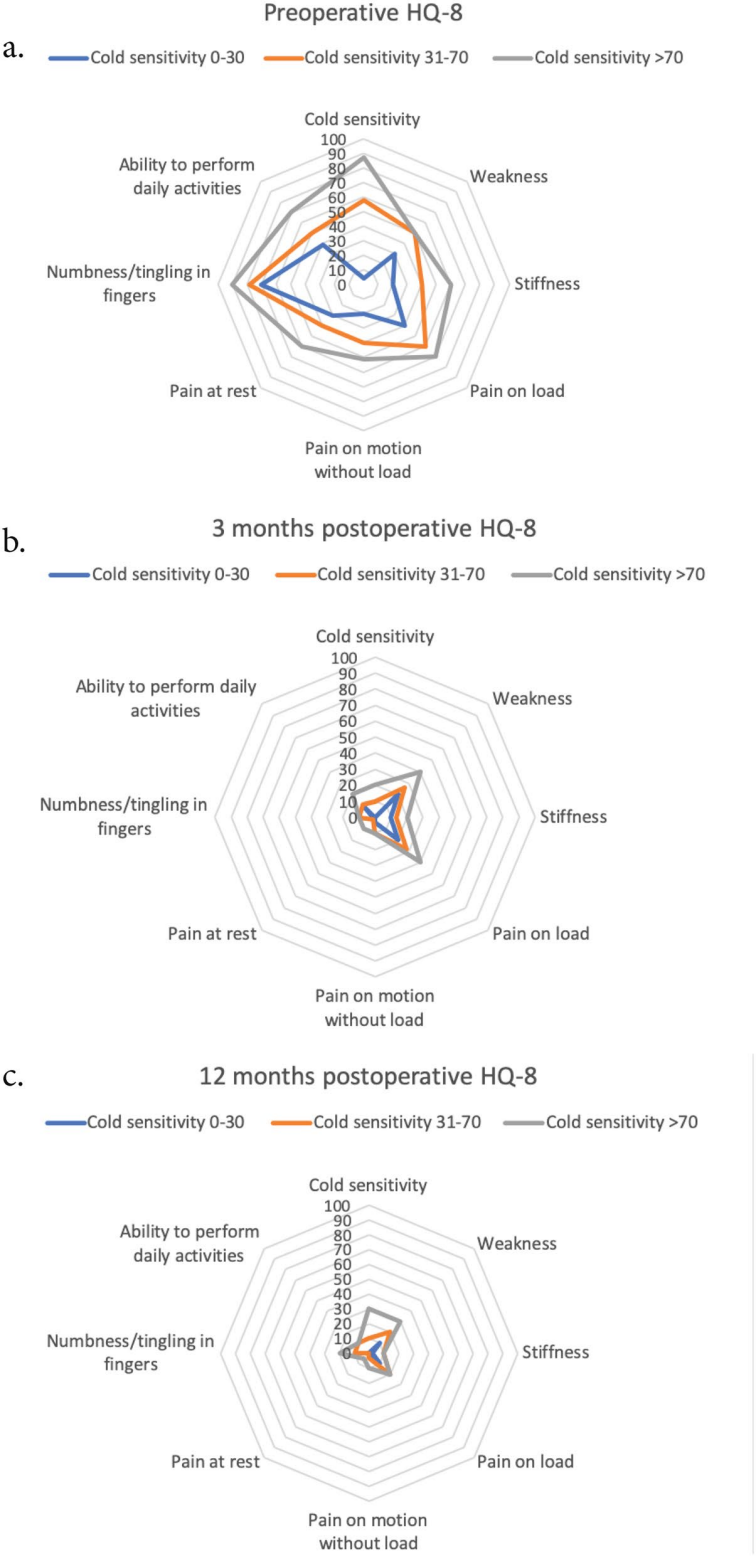
HQ-8 scores over time in patients with mild (scored as ≤ 30/100), moderate (scored as 31–70/100) and severe (scored as > 70/100) cold sensitivity with carpal tunnel syndrome (CTS) treated with open carpal tunnel release (OCTR). (**a**) Preoperatively, (**b**) 3 months postoperatively, (**c**) 12 months postoperatively.

Patients with moderate and severe cold sensitivity scored higher on QuickDASH at all time points compared to patients with mild cold sensitivity (Table 2 and Fig. 1). The improvement in QuickDASH (change from preoperative to 12 months postoperative) did not differ between groups. Patients with moderate and severe cold sensitivity also generally scored higher on all other HQ-8 items at all time points compared to patients with mild cold sensitivity (Table 3 and Fig. 2).

**Table 3:** HQ-8 scores in cases with mild (cold sensitivity symptom scoring ≤ 30), moderate (cold sensitivity symptom scoring 31–70) and severe (cold sensitivity symptom scoring > 70) cold sensitivity treated with open carpal tunnel release for carpal tunnel syndrome.

HQ-8 items	Preoperative			P-value	3 months postoperative			P-value	12 months postoperative			P-value
	Mild cold sensitivity (n = 1,332)	Moderate cold sensitivity (n = 1,072)	Severe cold sensitivity (n = 923)		Mild cold sensitivity (n = 472)	Moderate cold sensitivity (n = 353)	Severe cold sensitivity (n = 302)		Mild cold sensitivity (n = 350)	Moderate cold sensitivity (n = 270)	Severe cold sensitivity (n = 217)	
Cold sensitivity	4 [0–20]	58 [50–68]*	87 [80–95]*	**< 0.0001**	0 [0–10]	10 [0–30]*	20 [2–55]*	**< 0.0001**	0 [0–10]	10 [0–40]*	30 [1–70]*	**< 0.0001**
Weakness	30 [10–60]	50 [33–70]*	50 [70–83]*	**< 0.0001**	20 [10–40]	26 [10–50]	40 [11–70]*	**< 0.0001**	10 [0–30]	20 [0–44]	30 [1–60]*	**< 0.0001**
Stiffness	20 [0–44]	40 [20–60]*	60 [30–78]*	**< 0.0001**	10 [0–30]	13 [1–35]*	20 [9–41]	**< 0.0001**	2 [0–20]	10 [0–30]*	10 [0–50]*	**< 0.0001**
Pain on load	40 [10–65]	60 [37–72]*	70 [50–84]*	**< 0.0001**	20 [10–40]	28 [10–50]*	40 [17–70]*	**< 0.0001**	10 [0–25]	20 [0–40]*	20 [0–51]*	**< 0.0001**
Pain on motion without load	20 [1–40]	40 [20–60]*	51 [30–70]*	**< 0.0001**	3 [0–14]	10 [0–20]*	10 [1–40]*	**< 0.0001**	0 [0–10]	3 [0–20]*	10 [0–40]*	**< 0.0001**
Pain at rest	30 [8–60]	40 [20–70]*	60 [39–80]*	**< 0.0001**	0 [0–10]	2 [0–20]*	10 [0–30]*	**< 0.0001**	0 [0–10]	0 [0–20]*	5 [0–37]*	**< 0.0001**
Numbness/tingling in fingers	70 [50–83]	78 [60–90]*	90 [80–97]*	**< 0.0001**	1 [0–10]	10 [0–21]*	10 [0–50]*	**< 0.0001**	0 [0–20]	10 [0–30]*	20 [0–60]*	**< 0.0001**
Ability to perform daily activities	39 [17–60]	50 [36–70]*	70 [50–84]*	**< 0.0001**	10 [0–30]	11 [0–30]*	20 [4–56]*	**< 0.0001**	0 [0–20]	10 [0–30]*	10 [0–50]*	**< 0.0001**

Groups based on how patients scored the item regarding cold sensitivity in the preoperative HAKIR questionnaire 8 (HQ-8), scored 0–100. Numbers presented as median [interquartile range, IQR]. Kruskal–Wallis was used for significance testing.

Bold values indicate P-value < 0.05.

*Significant at < 0.05 level compared to the group to the left.

### Cold sensitivity and prediction of Quick DASH at three and 12 months

In the linear regression analysis, without adjustment for another surgical procedure in the hand/arm, compared to mild cold sensitivity, moderate cold sensitivity predicted higher QuickDASH scores at 3 months postoperatively by 3.6 points (95% CI 1.1–6.2; p = 0.006) and by 7.6 points (4.3–11.0; p < 0.0001) at 12 months postoperatively. Severe cold sensitivity predicted higher QuickDASH scores at 3 months postoperatively by 12.9 points (95% CI 10.2–15.6; p < 0.0001) and by 14.8 points (11.3–18.4; p < 0.0001) at 12 months postoperatively compared to mild cold sensitivity.

In a linear regression analysis, when adjusting for a concomitant ulnar nerve decompression, compared to mild cold sensitivity, moderate cold sensitivity predicted higher QuickDASH at 3 months postoperative with 3.6 points (1.0–6.2; p = 0.006), and severe cold sensitivity predicted higher QuickDASH at 3 months postoperative with 12.8 points (10.1–15.5; p < 0.0001). At 12 months postoperative, moderate cold sensitivity predicted higher QuickDASH scores with 7.3 points (4.0–10.6; p < 0.0001), and severe cold sensitivity predicted higher QuickDASH scores with 14.6 points (11.1–18.2; p < 0.0001; mild cold sensitivity was reference).

When adjusting for any other concomitant surgery in the hand/arm, compared to mild cold sensitivity, moderate cold sensitivity predicted higher QuickDASH at 3 months postoperative with 3.6 points (0.99–6.1; p = 0.007), and severe cold sensitivity predicted higher QuickDASH at 3 months postoperative with 12.8 (10.1–15.5; p < 0.0001). At 12 months postoperative, moderate cold sensitivity predicted higher QuickDASH scores with 7.6 points (4.3–10.9; p < 0.0001), and severe cold sensitivity predicted higher QuickDASH scores with 14.8 points (11.3–18.3; p < 0.0001; mild cold sensitivity was reference).

Analysis of cold sensitivity scoring as a continuous variable, without adjustment for another surgical procedure in the hand/arm, showed that a one-point increase in the cold sensitivity scoring preoperatively predicted increased postoperative QuickDASH scores at 12 months by 0.20 points (0.16–0.24; p < 0.0001).

### Cold sensitivity and prediction of change in total QuickDASH score

Moderate cold sensitivity had no statistically significant effect on change in QuickDASH score in the linear regression analysis (data not shown). Severe cold sensitivity predicted an increased change in total QuickDASH score from preoperatively to 12 months postoperatively by 3.7 points (0.72–6.60; p = 0.015). Analysis of cold sensitivity scoring as a continuous variable showed a one-point increase in the cold sensitivity scoring preoperatively increased the change in total QuickDASH score from preoperatively to 12 months postoperatively by 0.06 points (0.016–0.11; p = 0.008).

## Discussion

Cold sensitivity rapidly improved after OCTR, as did the other evaluated symptoms in the HQ-8 questionnaire. More importantly, patients who reported moderate or severe cold sensitivity before OCTR generally had more disability and worse cold sensitivity-related symptoms after surgery than patients who reported mild cold sensitivity. However, the relative improvement, as measured by the change in QuickDASH scores, did not differ between the groups.

The items in the HQ-8 are very similar to the items in the CISS questionnaire, which is a validated instrument for evaluating cold sensitivity^8^. Our results confirm that cold sensitivity is more associated with several other symptoms, as demonstrated by higher symptom scorings for pain, numbness, stiffness and weakness, among cases who scored their cold sensitivity as moderate or severe than among cases who scored their cold sensitivity as mild. One smaller previous study (n = 100) reported cold sensitivity in 52% of patients with CTS or ulnar nerve compression at the elbow^4^. Another study, using the Boston Carpal Tunnel Questionnaire, of the evaluated outcome after OCTR in 102 hands reported cold sensitivity, defined as colour changes or severe pain in the fingers after exposure to cold, in 46/102 (45%) cases. Cold sensitivity was associated with less improvement following surgery^9^. These results correspond well to our results, and the frequencies are similar to those seen after hand trauma^10,11^. In a normal population, cold sensitivity, defined as > 50 in CISS or by > 4 on a 10-point VAS (visual analogue scale), has been reported in 5–14%^10,12^, the higher prevalence being found in the northern parts of Sweden, where the climate is generally colder than in the south of the country. The present study population was geographically evenly distributed throughout Sweden, which strengthens the evaluation. It is possible that cold sensitivity becomes worse in winter, and slightly more patients in our study were operated on during the winter season. This is to be expected since there is less healthcare activity regarding elective cases during the summer season due to staff vacations. This might affect our results as patients may have experienced more cold sensitivity when scoring their symptoms during the winter season.

Several symptoms are related to cold sensitivity. All the symptoms investigated in the HQ-8 questionnaire were present in the patients with CTS and were improved by OCTR. Numbness and paraesthesia are the most prominent symptoms in patients with CTS, as our results illustrate. Interestingly, numbness was perceived as worse preoperatively in patients who rated their cold sensitivity as severe. This agrees with the results of a previous study, where numbness was found to be the most disturbing symptom of cold sensitivity^1^. In a population including patients with CTS and diabetes, treated with OCTR, cold sensitivity was reported as essentially the symptom that persisted the longest, compared to patients without diabetes. This was not seen in the present study^6^. Possible explanations include vasoregulatory imbalance, causing longer periods of vasoconstriction when exposed to cold, and the presence of diabetic small fibre neuropathy^13^.

The pathophysiology behind cold sensitivity is still not entirely understood. Patients with CTS have impaired cold detection thresholds, suggesting pathology in the Aδ-fibers^14^. There is probably also a vascular component in cold sensitivity. A normal response to cold exposure includes initial peripheral vasoconstriction, followed by vasodilation and then a new period of vasoconstriction. This response may be altered following hand trauma or nerve compression^15^. Nerve injury, including amputation, in the upper extremity is associated with Raynaud’s phenomenon and cold sensitivity^16^. Patients with a previous hand trauma reported that cold sensitivity negatively impacted their daily life^17^, but it improved over time^18^. We cannot, on the basis of the present results, shed detailed light on the pathophysiology of cold sensitivity in CTS and after OCTR. However, Aδ-fibers are most probably affected by the compression trauma^19^, particularly in the more severe CTS cases, and C-fibers may be influenced by sympathetic activity^20^. The scores for numbness and paraesthesia were higher among the patients with a cold sensitivity score > 30 at all time points and the QuickDASH scores were also higher among those patients with moderate or severe cold sensitivity. One interpretation of the results may be that patients with a more severe level of CTS, i.e. a higher risk of signs of nerve degeneration in the median nerve, more often experienced cold sensitivity. This remains to be explored in more detailed studies using electrophysiology staging of CTS, such as the presence of segmental demyelination, conduction block or axonal degeneration^21–23^. Such data are not available in the current national registry. Furthermore, data concerning concomitant co-morbidities, which may also be relevant, were not available.

New data suggest that better sensory recovery following surgical repair of peripheral nerve lesions in the upper extremity is associated with less cold sensitivity and less neuropathic pain^24^. It is possible that the same results could be obtained in patients who experience severe cold sensitivity in CTS with a targeted rehabilitation program after surgery.

Since we used “real world data” from the national registry for this study, a limited number of patients had undergone concomitant surgery in the hand/arm. In the three used linear regression models, i.e. with and without adjustment for concomitant surgical procedures in the hand/arm, we did not find any evidence that a concomitant ulnar nerve decompression affected the postoperative cold sensitivity experienced by the patients. Also, having had another hand surgical procedure performed at the same time as the carpal tunnel release, did not seem to affect cold sensitivity ratings. This could be valuable information for the surgeon treating multiple hand disorders at the same time.

## Conclusions

Cold sensitivity considerably improves after OCTR, regardless of preoperative severity. Preoperative moderate or severe cold sensitivity is associated with more disability and residual symptoms after OCTR, and adjustment for a concomitant ulnar nerve decompression or other concomitant surgical procedures in the hand/arm does not influence the results. This is important information which should be given to patients with CTS before surgery.

## Methods

### Study population

Data from OCTRs registered in the Swedish National Quality Registry for Hand Surgery (HAKIR; www.hakir.se) from 2010–2016 were used (identified through ICD-10 diagnosis code G560 and KKÅ97 operation code ACC51). HAKIR was set up in 2010 and all seven hand surgical university clinics in Sweden as well as two larger private units report to the registry. Patients provide informed consent before they are included in the registry. Data on diabetes status were retrieved from the Swedish National Diabetes Registry (www.ndr.se). Patients ≥ 18 years of age were included.

### Questionnaires

Patients received the Swedish version of the QuickDASH questionnaire (short version of the Disabilities of the Arm, Shoulder and Hand^25^) before and at three and 12 months after surgery, either as an online survey or by traditional mail. Patients received one reminder by text message two days after receiving the survey if they had not replied to it. The QuickDASH contains 11 items that are scored from 1 to 5. A total score from 0 to 100 is calculated, where 100 represents the most possible disability. The patients also received the HAKIR questionnaire 8 (the HQ-8 questionnaire is available at www.hakir.se) that is more symptom-specific and comprises eight Likert-scale questions, where each question is scored 0–100, with 100 representing the worst possible symptoms^26^. We defined severe cold sensitivity as a score of > 70, moderate cold sensitivity as a score of 30–70 and mild cold sensitivity as a score of < 30 on the question regarding cold sensitivity in the preoperative HQ-8. The cut-off of < 30 for mild cold sensitivity was based on the low occurrence of preoperative cold sensitivity in patients operated on for trigger finger, ganglion, de Quervain, Dupuytren’s contracture, and osteoarthritis of the first carpometacarpal joint, where cold sensitivity is not a prominent preoperative symptom, as presented in the HAKIR annual report^27^. Patients who answered the postoperative questionnaires were included in the calculations regardless of whether or not they had answered the preoperative questionnaires.

### Statistics

Normally distributed data are presented as mean ± standard deviation (SD). Skewed data are presented as median [interquartile range, IQR]. Nominal data are presented as a number (%). The Chi-squared test was used when comparing nominal data from two groups. The Kruskal–Wallis test with subsequent Bonferroni correction was used to compare groups. Changes over time were analysed using Friedman’s two-way analysis and adjusted with the Bonferroni correction for multiple testing. Linear regression analyses were used to study the effects of moderate and severe cold sensitivity on postoperative QuickDASH scores, adjusted for age at surgery, gender and diabetes in three different models with (a) the original “real world data”; and with adjustment for (b) a concomitant ulnar nerve decompression or (c) other concomitant surgical procedures in the hand/arm. IBM SPSS Statistics version 24 (SPSS Inc., Chicago, IL, USA) was used for all calculations. Spider diagrams were designed using Microsoft Excel for Mac, version 16.30 (Microsoft). Each operated hand was considered a separate statistical entity. A p-value of < 0.05 was considered statistically significant.

### Ethics approval and consent to participate

This study was approved by the Regional Ethical Review Board in Lund, Sweden (2016/931, 2018/57 and 2018/72). We confirm that we have read the Journal’s position on issues involved in ethical publication and affirm that this report is consistent with those guidelines. The research was performed according to the Helsinki Declaration.
